# SP600125, a JNK-Specific Inhibitor, Regulates *in vitro* Auricular Cartilage Regeneration by Promoting Cell Proliferation and Inhibiting Extracellular Matrix Metabolism

**DOI:** 10.3389/fcell.2021.630678

**Published:** 2021-03-16

**Authors:** Peiling Zhang, Yanqun Liu, Litao Jia, Zheng Ci, Wei Zhang, Yu Liu, Jie Chen, Yilin Cao, Guangdong Zhou

**Affiliations:** ^1^Department of Plastic and Reconstructive Surgery, Shanghai Key Laboratory of Tissue Engineering, Shanghai Ninth People’s Hospital, Shanghai Jiao Tong University School of Medicine, Shanghai, China; ^2^Research Institute of Plastic Surgery, Wei Fang Medical College, Wei Fang, China; ^3^National Tissue Engineering Center of China, Shanghai, China; ^4^Department of Anesthesiology, Shanghai Ninth People’s Hospital, Shanghai Jiao Tong University School of Medicine, Shanghai, China

**Keywords:** JNK inhibitors, SP600125, cell proliferation, extracellular matrix, *in vitro*, cartilage regeneration

## Abstract

*In vitro* construction is a major trend involved in cartilage regeneration and repair. Satisfactory *in vitro* cartilage regeneration depends on a suitable culture system. Current chondrogenic culture systems with a high content of transforming growth factor beta-1 effectively promote cartilaginous extracellular matrix (ECM) production but inhibit chondrocyte survival. As is known, inhibition of the c-Jun *N*-terminal kinase (JNK) signaling pathway acts in blocking the progression of osteoarthritis by reducing chondrocyte apoptosis and cartilage destruction. However, whether inhibiting JNK signaling resists the inhibitory effect of current chondrogenic medium (CM) on cell survival and affects *in vitro* auricular cartilage regeneration (including cell proliferation, ECM synthesis, and degradation) has not been investigated. In order to address these issues and optimize the chondrogenic culture system, we generated a three-dimensional *in vitro* auricular cartilage regeneration model to investigate the effects of SP600125 (a JNK-specific inhibitor) on chondrocyte proliferation and ECM metabolism. SP600125 supplementation efficiently promoted cell proliferation at both cellular and tissue levels and canceled the negative effect of our chondrogenic culture system on cell survival. Moreover, it significantly inhibited ECM degradation by reducing the expressions of tumor necrosis factor-alpha, interleukin-1-beta, and matrix metalloproteinase 13. In addition, SP600125 inhibited ECM synthesis at both cellular and tissue levels, but this could be canceled and even reversed by adding chondrogenic factors; yet this enabled a sufficient number of chondrocytes to be retained at the same time. Thus, SP600125 had a positive effect on *in vitro* auricular cartilage regeneration in terms of cell proliferation and ECM degradation but a negative effect on ECM synthesis, which could be reversed by adding CM. Therefore, a combination of SP600125 and CM might help in optimizing current chondrogenic culture systems and achieve satisfactory *in vitro* cartilage regeneration by promoting cell proliferation, reducing ECM degradation, and enhancing ECM synthesis.

## Introduction

Cartilaginous defects are among the most common human diseases. Damaged cartilage has poor self-repair capacity because the cartilaginous extracellular matrix (ECM) is neither vascularized nor innervated (Dhinsa and Adesida, 2012; Yin et al., 2016). To treat cartilage damage effectively, the defective area needs to be filled with repair tissue (Fini et al., 2013). Cartilage tissue engineering, which can regenerate functional tissues similar to native ones, is a promising strategy for repairing damaged cartilage (Zheng et al., 2014; He et al., 2018). *In vitro* construction has been an important direction and trend for cartilage regeneration because of its many advantages in avoiding cell leakage, reducing inflammatory reactions triggered by tissue culture scaffolds, and maintaining stability after transplantation *in vivo* (He et al., 2017), which are all of great significance for clinical applications.

To achieve satisfactory cartilage regeneration *in vitro*, a suitable chondrogenic culture system is needed. The chondrogenic medium (CM) currently used for cartilage regeneration contains a high content of transforming growth factor beta-1 (TGF-β1), which can effectively improve *in vitro* cartilage regeneration by promoting chondrocytes or cells with chondrogenic potential such as bone marrow stromal cells to generate abundant cartilaginous ECM (Blunk et al., 2002; Yang and Barabino, 2013). However, overexpression of TGF-β1 was reported to induce chondrocyte apoptosis and to reduce the number of active cells (Fang et al., 2016; Zhang et al., 2018), which is obviously unfavorable for the stability and maintenance of regenerated cartilage after implantation *in vivo*.

Previous studies have indicated that inhibition of c-Jun *N*-terminal kinase (JNK), part of a highly complex signaling pathway, could reduce chondrocyte apoptosis remarkably in cases of osteoarthritis (OA; Weston and Davis, 2007; Lu et al., 2014; Yang et al., 2016). This might provide a strategy to overcome the negative effect of current chondrogenic culture systems on cell survival. More importantly, accumulating evidence indicates that inhibition of the JNK pathway can effectively reduce ECM degradation associated with OA (Chen et al., 2016; Cheng et al., 2016; Ge et al., 2017), which is obviously beneficial for ECM deposition in regenerated cartilage. Nevertheless, all conclusions on the effects of the JNK inhibitors mentioned above were based on cellular levels or the model of OA being used. Whether JNK inhibitors have the same effects on *in vitro* auricular cartilage regeneration in terms of enhancing cell survival and decreasing ECM degradation has not yet been investigated. To determine the effects of JNK inhibitors on *in vitro* auricular cartilage regeneration (including cell proliferation, ECM synthesis, and degradation) and thus optimize current chondrogenic culture systems, several issues should be clarified. First, can JNK inhibitors promote cell proliferation so as to enhance the number of active cells in three-dimensional (3D) cartilage regeneration models *in vitro*? Second, can the use of JNK inhibitors help resist or even reverse the negative effect of CM on chondrocyte survival? Third, what effects do JNK inhibitors have on ECM metabolism during *in vitro* cartilage regeneration?

To address these problems, we established an *in vitro* 3D auricular cartilage regeneration model. Rabbit auricular chondrocytes were seeded into polyglycolic acid/polylactic acid (PGA/PLA) scaffolds and then cultured with or without SP600125 (a JNK-specific inhibitor) in either regular medium (RM) or CM for 8 weeks. Based on this, a series of evaluations were conducted to clarify the effects of SP600125 on cell proliferation, ECM metabolism, and cartilage regeneration, to help in optimizing the chondrogenic culture system.

## Materials and Methods

### Isolation and Culture of Auricular Chondrocytes

Auricular cartilage was obtained from New Zealand White rabbits, purchased from the Shanghai Jia Gan Experimental Animal Raising Farm (Shanghai, China). All animal study protocols were approved by the Animal Care and Experiment Committee of Shanghai Jiao Tong University School of Medicine. After the perichondrium had been removed, cartilage slices were minced into 1.0-mm^3^ pieces, washed in phosphate-buffered saline (PBS) containing 1% penicillin–streptomycin (Gibco, Grand Island, NY, United States), and then treated with 0.15% collagenase II (Gibco) in Dulbecco’s Modified Eagle’s Medium (DMEM; Gibco) for 8–12 h at 37°C (Rodriguez et al., 1999). Isolated chondrocytes were then seeded into 10-cm culture dishes in DMEM containing 10% fetal bovine serum (FBS; HyClone, Logan, UT, United States) and 1% penicillin–streptomycin, and placed in an incubator at 37°C with 95% humidity and 5% CO_2_. Chondrocytes from the first and second passages were used for subsequent experiments.

### Cell Counting Kit-8 Assay of Cell Proliferation

Harvested chondrocytes from the first passage were seeded at a density of 2 × 10^3^ cells/well in 96-well plates. After incubation at 37°C for 24 h, chondrocytes were cultured in DMEM with 10% FBS supplemented with or without 0.02 mg/ml of the JNK pathway inhibitor SP600125 (AdooQ Bioscience, Irvine, CA, United States). The two groups were assessed individually for cell proliferation using cell counting kit-8 (CCK-8) kits (Dojindo Laboratories, Kumamoto, Japan) on days 1, 3, 5, and 7 as described (Zhang et al., 2014).

### Counting of Chondrocytes

Cell yields following different treatments were measured by cell counting. Chondrocytes from the first passage were harvested and seeded into 24-well plates at a density of 1.0 × 10^4^ cells/ml (1 ml per well). After incubation at 37°C for 24 h, chondrocytes were cultured in medium with (+) or without (-) SP600125. Cells were harvested and counted on days 1, 3, 5, and 7.

### Preparation and *in vitro* Culture of Cell-Scaffold Constructs

Thirty micrograms of unwoven PGA fibers (National Tissue Engineering Center of China, Shanghai, China) was compressed into cylindrical scaffolds 9 mm in diameter and 2 mm thick. Next, 1.0% PLA (Sigma-Aldrich, St. Louis, MO, United States) in dichloromethane solvent was added to solidify the PGA scaffolds. The properties of these PGA/PLA scaffolds have been reported in detail in many previous studies (Liu et al., 2016; Chen et al., 2017). The scaffolds were then disinfected with 75% ethanol for 40 min and washed three times with PBS. Harvested chondrocytes from the second passage were seeded into each scaffold (0.1 ml per scaffold) at a concentration of 6.0 × 10^7^ cells/ml in RM (DMEM containing 10% FBS), followed by a 4-h incubation, according to a previously described method to form cell-scaffold constructs (Liu et al., 2008; Yan et al., 2009). Next, half of the cell–scaffold constructs were cultured in RM with (+) or without (-) 0.02 mg/ml SP600125, while the other half were cultured in CM: DMEM containing 10 ng/ml of TGF-β1 (R&D Systems, Minneapolis, MN, United States), 40 ng/ml of dexamethasone (Sigma-Aldrich), 100 ng/ml of insulin-like growth factor (IGF)-I (R&D Systems), and other supplements (Li et al., 2019) with (+) or without (−) 0.02 mg/ml of SP600125. All constructs were cultured at 37°C in 95% humidity with 5% CO_2_ for 8 weeks.

### Cell Adhesion

After 24 h of incubation, the cell-scaffold samples were transferred into new six-well plates for subsequent culture. The remaining cells in the primer six-well plate were collected and counted. Cell seeding efficiencies of scaffolds with different media contents were calculated based on the following formula:

(total cell number - remaining cell number)/total cell number × 100% (Liu et al., 2010).

### Scanning Electron Microscopy

Extracellular matrix production on the surfaces of samples from different groups was observed by SEM (Philips XL-30, Amsterdam, Netherlands) after culture *in vitro* for 1, 3, 7, and 14 days. Samples were washed three times with PBS and fixed overnight in 2.5% glutaraldehyde at 4°C. After dehydration in a graded series of ethanol solution, samples were coated with gold and examined using SEM (Xu et al., 2017).

### Wet Weight, Volume, and Thickness

After 8 weeks of *in vitro* culture, samples from all four groups were collected and weighed using an electronic balance. The thickness of each sample was measured with a Vernier caliper.

### Histological and Immunohistochemical Evaluations

Tissue samples were fixed in 4% paraformaldehyde for 48 h before being embedded in paraffin wax and 5-μm sections were prepared. To evaluate the structure, ECM deposition, and chondrogenic differentiation of engineered tissues, sections were stained with hematoxylin and eosin (HE), Safranin O (SO), toluidine blue (TB), and type II collagen (COL II; mouse anti-human COL II monoclonal antibody, 1:100, Santa Cruz Biotechnology, Dallas, TX, United States), as described (Liu et al., 2008).

### Quantitative Reverse Transcription Polymerase Chain Reaction Analysis

Total RNA was extracted from both cell and tissue samples (*n* = 3 per group) of different groups, and cDNA was obtained by reverse transcription (RT) according to a previously described method (Jiang et al., 2010). RT–quantitative reverse transcription polymerase chain reaction (RT-qPCR) was performed according to the manufacturer’s protocol (Thermo Fisher Scientific, Waltham, MA, United States). The expression levels of the genes *TGF-*β*1*, *IGF*, *Aggrecan*, *COLIIA1*, *Sox9*, *TNF-*α, *IL-1*β, and *MMP13* were analyzed. The housekeeping gene encoding *B-actin* was quantified as an internal control. Forward and reverse primer sequences are listed in Supplementary Table 1. Expression levels were analyzed using the 2^–Δ^ ^Δ^ CT method, as described (Livak and Schmittgen, 2001).

### Biomechanical and Biochemical Evaluations

Young’s modulus of samples in different groups (*n* = 6 per group) was measured using a biomechanical analyzer (Instron-5542, Canton, MA, United States), as described (Chen et al., 2017). A constant compressive strain at 0.5 mm/min was applied until 80% of maximal deformation was reached, and the first 40% was used to plot the stress–strain curves. Young’s modulus was calculated according to stress–strain curves for statistical analysis.

After this biomechanical analysis, samples in the different groups were weighed and minced for DNA, total glycosaminoglycan (GAG), and total collagen quantifications. Quant-iT PicoGreen dsDNA assays (Invitrogen, Carlsbad, CA, United States) were used to quantify DNA contents of samples after culture *in vitro* for 3, 7, or 14 days and 8 weeks. Samples were treated and analyzed as described (Schagemann et al., 2010). GAG contents of tissue-engineered cartilage were quantified by a dimethylmethylene blue chloride (DMMB; Sigma-Aldrich) method, as described (Enobakhare et al., 1996). Total collagen contents of samples were quantified by a hydroxyproline assay, as described (Reddy and Enwemeka, 1996).

### Statistical Analysis

All values are expressed as the mean ± standard deviation (SD). Statistical significance was analyzed by independent Student’s *t*-tests and one-way analysis of variance followed by *post hoc* tests with the Student–Newman–Keuls (SNK) method for comparison between groups using IBM SPSS Statistics (v. 25; IBM Corp., Armonk, NY, United States). A *p*-value less than 0.05 was considered to be statistically significant.

## Results

### Influence of SP600125 on Chondrocyte Activity and Function

**FIGURE 1:**
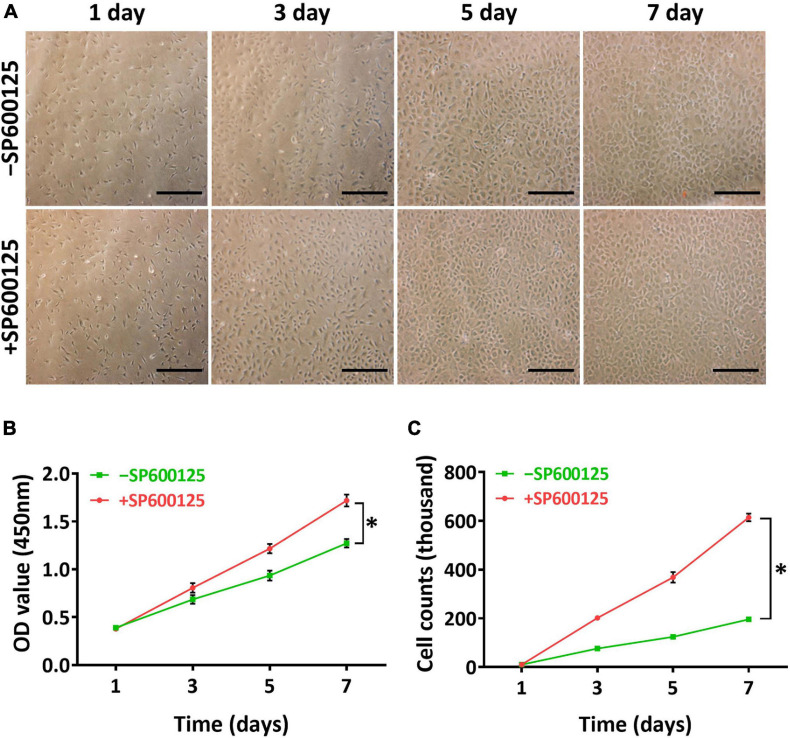
Influence of SP600125 on auricular chondrocyte proliferation. Chondrocyte proliferation was observed by light microscopy **(A)**, CCK-8 **(B)**, and cell counts **(C)**. The + SP600125 culture system promoted the proliferation of chondrocytes compared with the -SP600125 culture system as shown consistently by light microscopy, CCK-8 assays, and cell counts. Scale bars = 50 μm. “*”indicates statistical significance (*p* < 0.05). CCK-8, cell counting kit 8; JNK, c-Jun *N*-terminal kinase.

A two-dimensional (2D) model was constructed to study the short-term effects of SP600125 on chondrocytes. Light microscopy observations revealed that there were more active chondrocytes in the + SP600125 group compared with the -SP600125 group per field of vision (Figure 1A). Furthermore, CCK-8 cell proliferation curves showed differences between the two groups (Figure 1B), and cell counting identified an increased number of chondrocytes in the + SP600125 group compared with the -SP600125 group (Figure 1C). These results indicate that SP600125 promoted the proliferation of chondrocytes.

**FIGURE 2:**
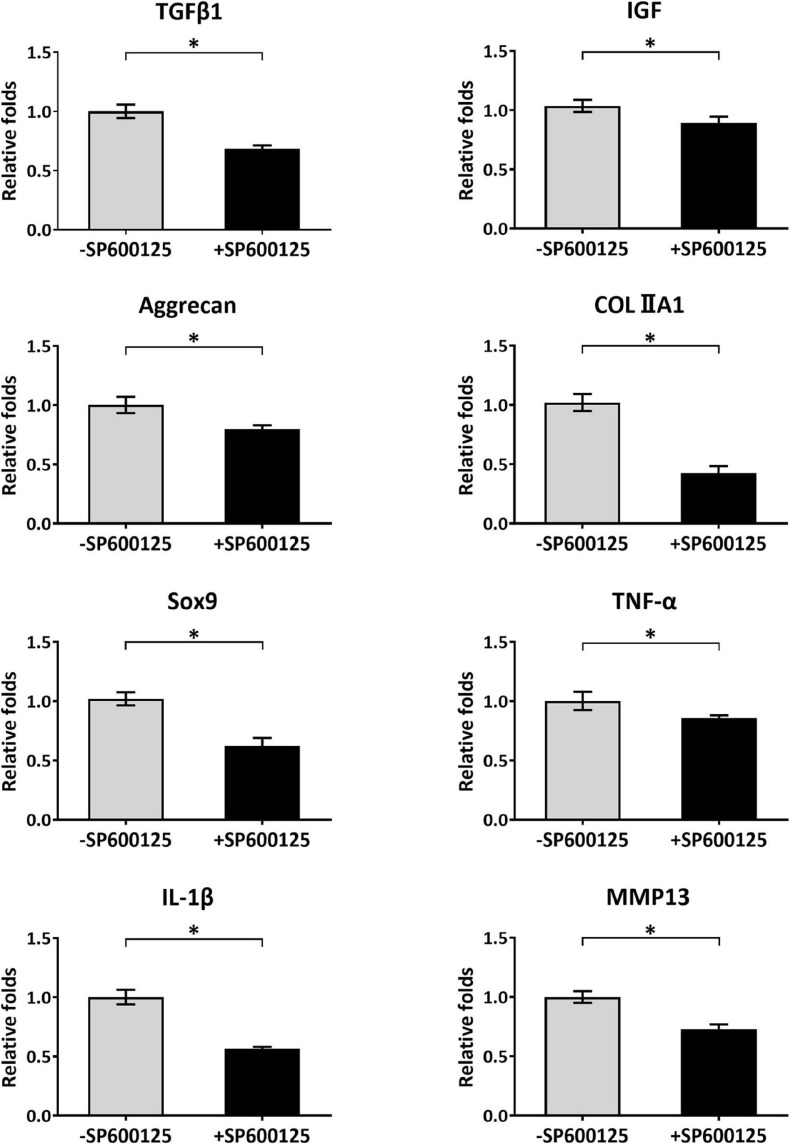
Analysis of cartilage-related and inflammatory-related genes of auricular chondrocytes. After treatment with SP600125 for 24 h, the expressions of cartilage-related genes (*TGF-*β*1*, *IGF*, *Aggrecan*, *COLIIA1*, and *Sox9*) involved in the synthesis of ECM were downregulated. Simultaneously, the expression levels of inflammatory-related genes (*TNF-*α and *IL-1*β) and *MMP13*, which are involved in ECM catabolism, were significantly downregulated. “*”indicates statistical significance (*p* < 0.05). ECM, extracellular matrix; MMP13, matrix metalloproteinase 13.

Cartilage-related genes and inflammatory-related genes were analyzed by RT-qPCR to evaluate the short-term effects of SP600125 on chondrocytes (Figure 2). After treatment with SP600125 for 24 h, expression levels of inflammatory-related genes such as those encoding *TNF-*α and *IL-1*β were decreased. Meanwhile, the expression of *MMP13* was significantly downregulated probably in response to decreased expression of the genes for *TNF-*α and *IL-1*β, which suggests inhibited degradation of cartilaginous ECM. Unexpectedly, the expressions of some cartilage-related genes, such as *TFG*β*1*, *IGF*, *Aggrecan*, *COLIIA1*, and *Sox9* were downregulated, which indicates an inhibitory effect on ECM synthesis. These results demonstrate that SP600125 had an inhibitory effect on both ECM synthesis and degradation.

### Biocompatibility of Cell-Scaffold Constructs in a Three-Dimensional Culture System

**FIGURE 3:**
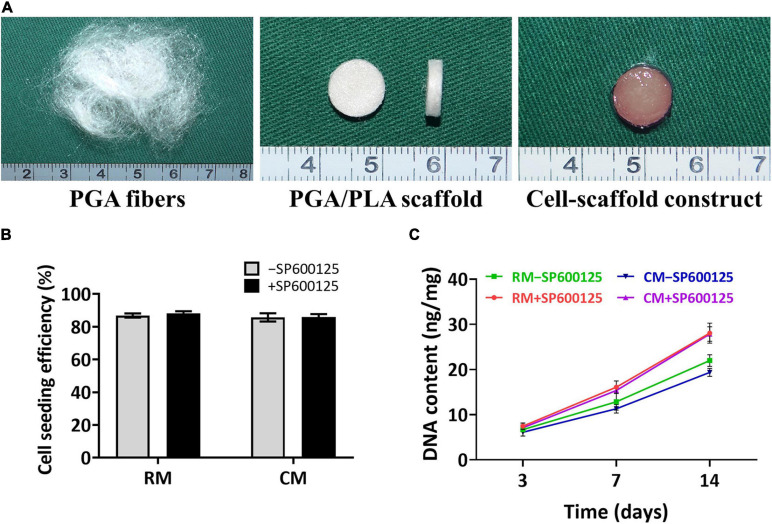
Preparation of *in vitro* constructed cartilage and biocompatibility of scaffolds. Unwoven PGA fibers were compressed into PGA/PLA scaffolds 9 mm in diameter and 2 mm thick, and then rabbit auricular chondrocytes were seeded into each scaffold to form cell-scaffold constructs **(A)**. There were no significantly differences in cell seeding efficiencies among each group **(B)**. DNA content **(C)** of each group was enhanced with culture time. Treatment with SP600125 resulted in an increase of DNA content, while treatment with CM inhibited the growth of DNA content. However, the CM + SP600125 group showed that adding SP600125 reversed this inhibitory effect of CM and resulted in more DNA content. PGA, polyglycolic acid; PLA, polylactic acid; RM, regular medium; and CM, chondrogenic medium.

Thirty micrograms of unwoven PGA fibers was compressed into a cylinder 9 mm in diameter and 2 mm thick. Scaffolds were then solidified by PLA in dichloromethane solvent. Aliquots of 6.0 × 10^7^ chondrocytes were seeded onto each scaffold and cultured with or without SP600125 in either RM or CM (Figure 3A). Differences in cell seeding efficiency were not statistically significant (Figure 3B), which indicates no statistical difference in the initial number of cells among different groups. The DNA contents of all groups increased with culture times. Notably, the +SP600125 groups showed a more obvious growth trend, which indicates that cell proliferation was promoted in this 3D cartilage regeneration model (Figure 3C). The CM-SP600125 group showed lower levels of DNA than the RM-SP600125 group, which suggests an inhibitory effect of CM on cell proliferation. However, the CM + SP600125 group reversed the negative effect of CM on cell survival by showing a greater DNA content than in the RM-SP600125 group, which indicates a greater number of active cells. These results indicate that SP600125 supplementation promoted the proliferation of chondrocytes and reversed the negative effect of CM on cell survival.

**FIGURE 4:**
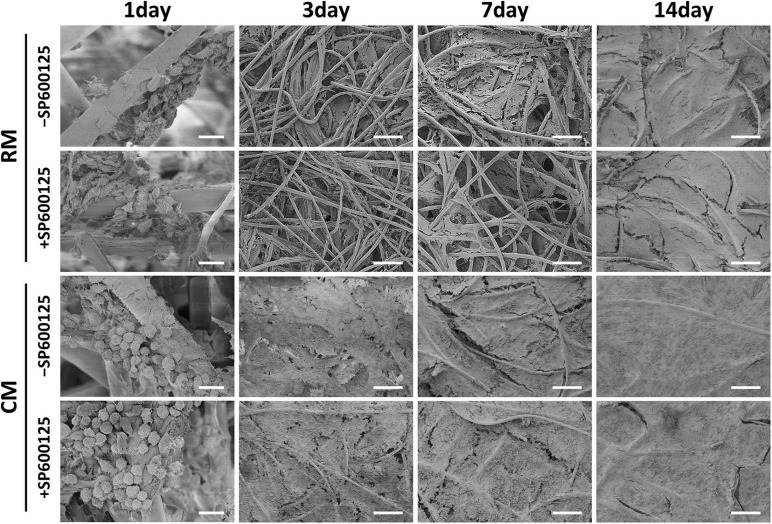
ECM production by auricular chondrocytes on PGA/PLA scaffolds observed using SEM. Chondrocytes on scaffolds were separated and displayed round shapes within 24 h. In the following culture period, the ECM deposition increased with time. Treatment with CM enhanced the ECM deposition, while treatment with SP600125 alone decreased it. However, the CM + SP600125 group showed that adding CM reversed this inhibitory effect of SP600125 alone and produced more ECM deposition. Scale bars (1 day) = 20 μm. Scale bars (3, 7, and 14 days) = 200 μm. ECM, extracellular matrix; PGA, polyglycolic acid; PLA, polylactic acid; RM, regular medium; and CM, chondrogenic medium.

The deposition of ECM was evaluated by SEM during the early stage of *in vitro* cartilage regeneration (Figure 4). SEM revealed that chondrocytes remained round and separated in the first 24 h, and there was no significant difference in morphology among the groups. After 3 days, the chondrocytes spread and began to secrete ECM to adhere to fibers. The addition of CM obviously accelerated and increased the deposition of cartilage ECM. At 7 days, more ECM was produced by chondrocytes, and the fiber interspaces were almost fully covered. At 14 days, the PGA fibers had been completely covered by abundant ECM. Less ECM deposition was observed in the RM + SP600125 group than in the RM-SP600125 group, which indicates inhibition of SP600125 on ECM synthesis. However, the CM + SP600125 group reversed this tendency by achieving obviously greater ECM deposition than in the RM + SP600125 and RM-SP600125 group. These results indicate that SP600125 inhibited ECM synthesis, but the addition of CM reversed this.

### Tissue-Engineered Cartilage Regeneration *in vitro*

#### Gross View and Histology of *in vitro* Cartilage Regeneration

**FIGURE 5:**
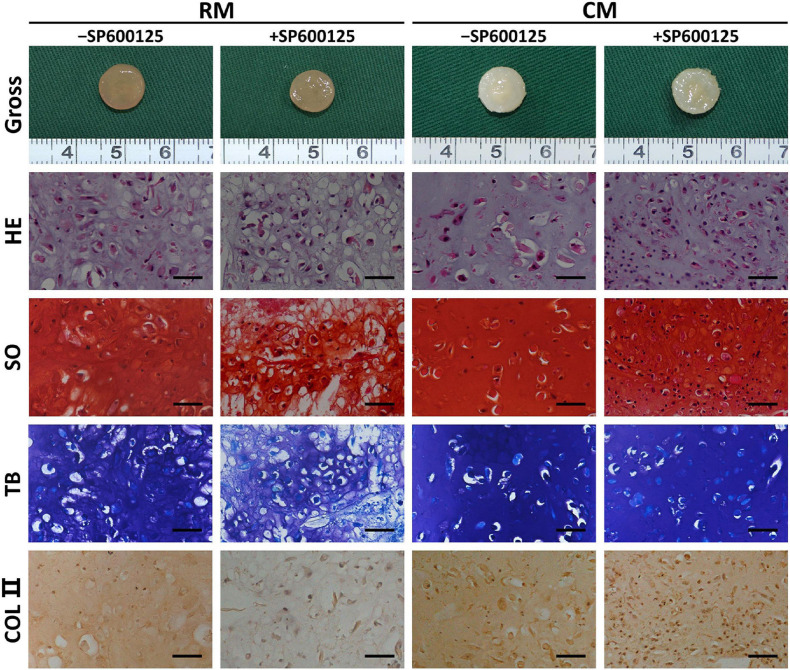
Gross view and histology of *in vitro* cartilage regeneration. Samples in different groups showed different colors and textures. The CM groups showed more mature cartilaginous tissue and increased ECM production than did the RM groups. Treatment with SP600125 alone decreased the ECM deposition and cartilage maturity with weak staining for cartilage-specific ECM molecules. However, the CM + SP600125 culture reversed the effect of SP600125 alone in reducing ECM synthesis and produced more active cells with stained nuclei compared with the -SP600125 groups at the same time. Scale bars = 50 μm. SO, Safranin O staining; TB, toluidine blue staining; COL II, type II collagen staining; RM, regular medium; CM, chondrogenic medium; and ECM, extracellular matrix.

After 8 weeks of *in vitro* culture, different groups appeared to have different gross appearances (Figure 5). The RM groups had a faint yellow appearance, while the CM groups showed a cartilaginous ivory-white appearance. The RM-SP600125 group showed a relatively smooth surface, while the RM + SP600125 group showed a more irregular one; however, differences between the cartilage in the CM + SP600125 and CM-SP600125 groups could not be distinguished by eye. Histology further supported the observed macroscopic results (Figure 5). Generally, the +SP600125 groups exhibited less ECM deposition and chondrogenic differentiation but more nucleated cells. The CM groups showed more mature cartilaginous tissue, increased ECM production, and better chondrogenic differentiation than did the RM groups, likely reflecting active chondrogenesis. In the RM-SP600125 group, some lacuna-like structures were observed with positive SO, TB, and COL II staining results. In the RM + SP600125 group, only undegraded PGA fibers and fibrous tissue were observed without formation of lacuna-like structures; and the SO, TB, and COL II staining results were extremely weak. The CM + SP600125 group showed more mature cartilaginous tissue with increased ECM deposition and better chondrogenesis in terms of cartilaginous features and thicknesses than did the RM-SP600125 group, which indicates that the addition of CM reversed the inhibitory effect of SP600125 on ECM synthesis. Moreover, more cells were obvious in the CM + SP600125 group than in the RM-SP600125 and CM-SP600125 groups, which suggests that the negative effect of CM on cell survival was reversed by the addition of SP600125. These results indicate that SP600125 inhibited ECM synthesis, but the addition of CM reversed this. At the same time, SP600125 reversed the negative effect of CM on cell survival by enhancing the numbers of active cells.

#### Quantitative Evaluations of *in vitro* Cartilage Regeneration

**FIGURE 6:**
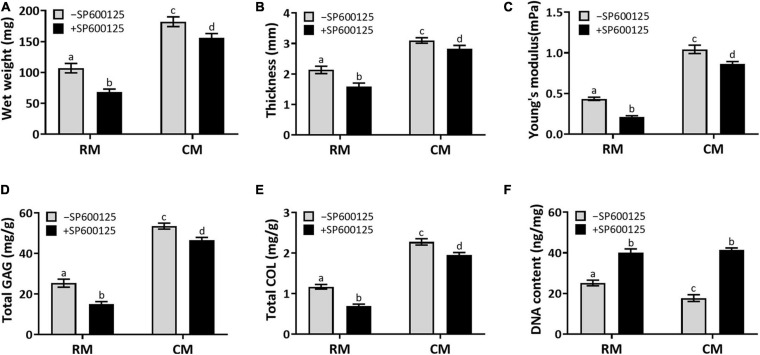
Wet weight, thickness, biomechanical, and biochemical evaluations. Wet weight **(A)**, thickness **(B)**, Young’s modulus **(C)**, total GAG **(D)**, and total COL **(E)** contents showed decreasing trends in the RM + SP600125 group compared with the RM-SP600125 group. However, the CM + SP600125 culture reversed these inhibitory effects. Treatment with SP600125 enhanced the DNA contents **(F)**, which indicates greater cell numbers. More importantly, the CM + SP600125 culture reversed the inhibitory effect of CM on cell survival. Columns with the same letters indicate no statistically significant differences (*p* > 0.05), whereas the columns with different letters indicate statistically significant differences (*p* < 0.05). ECM, extracellular matrix; RM, regular medium; CM, chondrogenic medium; GAG, glycosaminoglycan; and COL, collagen.

Quantitative examinations of cartilage ECM, such as wet weight, thickness, Young’s modulus, total GAG, and total COL contents (Figures 6A–E), were decreased in the +SP600125 groups. However, differences decreased from absolute changes of 40–50% in the RM groups to 10–20% in the CM groups, which indicates that SP600125 inhibited ECM synthesis during *in vitro* chondrocyte culture but that this was diminished by the addition of CM. More importantly, the amounts of ECM were increased in the CM + SP600125 group compared with the RM-SP600125 group, which indicates that combination of SP600125 and CM reversed the inhibitory effect of SP600125 on ECM synthesis. The DNA contents of the +SP600125 groups were much higher than in the -SP600125 groups, which indicates the promotion of cell proliferation (Figure 6F). The CM-SP600125 group showed less DNA content than in the RM-SP600125 group, which indicates the negative effect of CM on cell survival. However, the addition of SP600125 to CM reversed this negative effect on cell survival and produced greater DNA contents compared with the RM-SP600125 group. These results indicate that SP600125 inhibited ECM synthesis, but the addition of CM reversed this negative effect. At the same time, SP600125 reversed the negative effect of CM on cell survival by enhancing the number of active cells.

#### Characteristic Gene Expression of *in vitro* Cartilage Regeneration

**FIGURE 7:**
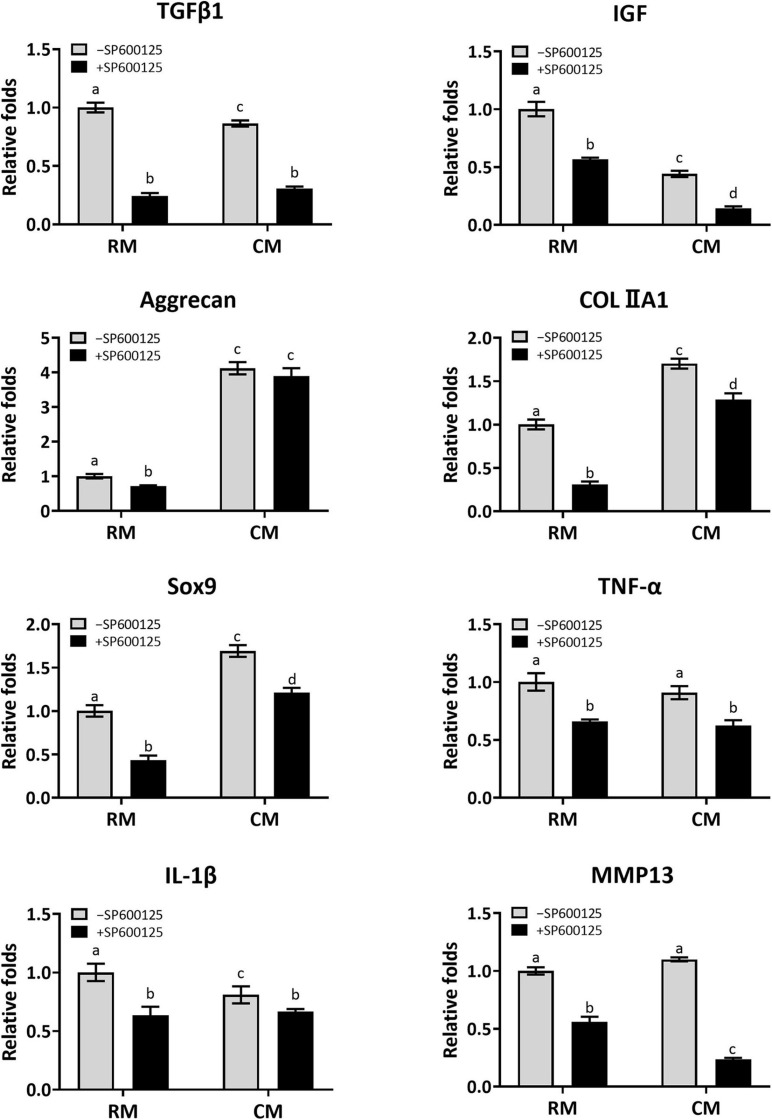
Analysis of the expression levels of cartilage-related genes and inflammatory-related genes of *in vitro* cultured cartilages. After the samples were cultured *in vitro* for 8 weeks, the expressions of cartilage-related genes (*TGF-*β*1*, *IGF*, *Aggrecan*, *COLIIA1*, and *Sox9*) were greatly reduced when treated with SP600125 alone. However, the CM + SP600125 culture reversed the inhibitory effect of SP600125 alone on ECM synthesis. The expressions of *TGF-*β*1* and *IGF* were also downregulated in the CM-SP600125 group, which was probably caused by feedback inhibition with the high contents of TGF-β1 and IGF in CM. Expressions of inflammatory-related genes (*TNF-*α and *IL-1*β) and of *MMP13*, which are involved in ECM catabolism, were downregulated in the + SP600125 groups. Columns with the same letters indicate no statistically significant differences (*p* > 0.05), whereas the columns with different letters indicate statistically significant differences (*p* < 0.05). RM, regular medium; CM, chondrogenic medium; ECM, extracellular matrix; and MMP13, matrix metalloproteinase 13.

Cartilage-related genes and inflammatory-related genes were analyzed by RT-qPCR to further evaluate the effect of SP600125 on this pathway and on cartilage formation (Figure 7). The expression levels of cartilage formation-related genes, such as *TGF-*β*1*, *IGF*, *Aggrecan*, *COLIIA1*, and *Sox9*, were decreased in the +SP600125 groups compared with the -SP600125 groups. However, the addition of CM obviously diminished these differences. Moreover, higher expression levels of cartilage-related genes were observed in the CM + SP600125 group compared with the RM-SP600125 group, which indicates that a combination of SP600125 and CM reversed the inhibitory effect of SP600125 on ECM synthesis. It is noteworthy that expression levels of *TGF-*β*1* and *IGF* were also decreased in the CM-SP600125 group, which was probably caused by feedback inhibition from the high concentrations of TGF-β1 and IGF-1 in CM. In addition, expressions of gene encoding *TNF-*α, *IL-1*β, and *MMP13* were decreased in the +SP600125 groups, which indicates reduction of inflammatory responses and ECM degradation. All these results indicate that SP600125 decreased ECM synthesis and degradation *in vitro*, but the inhibitory effect of SP600125 on ECM synthesis was reversed by the addition of CM.

## Discussion

Cartilage defects and degeneration remain difficult clinical problems because of the poor self-repair capacity of this connective tissue (Dhinsa and Adesida, 2012; Fini et al., 2013; Yin et al., 2016). *In vitro* construction is one of the major trends for cartilage regeneration. Although the chondrogenic culture systems used currently help in promoting ECM production, they inhibit chondrocyte survival, resulting in a sharp decrease in the number of active cells, which is obviously unfavorable for the stability and maintenance of regenerated cartilage after implantation *in vivo*. Here, we found that supplementation with SP600125 could significantly promote cell proliferation at both cellular and tissue levels and even cancel out the negative effect of CM on cell survival and thus effectively increase the number of active cells in regenerated cartilage. Moreover, SP600125 supplementation reduced ECM degradation by inhibiting the expression of the genes encoding *TNF-*α, *IL-1*β, and *MMP13*, which was also beneficial for the maintenance of regenerated cartilage. In addition, the inhibitory effect of SP600125 on ECM synthesis could be canceled and even reversed by adding chondrogenic factors. Therefore, the combination of SP600125 and CM could efficiently promote cell proliferation, reduce ECM degradation, improve ECM synthesis, and thus help in optimizing our chondrogenic culture system and achieve satisfactory *in vitro* cartilage regeneration.

Satisfactory cartilage regeneration *in vitro* depends on the numbers of active cells and the amount of ECM (Yokoo et al., 2005; Huey et al., 2012; Oseni et al., 2012). Chondrocytes are the only cell type in cartilage tissues and are thought to be involved in the maintenance of tissue homeostasis (Jiang and Tuan, 2015). Although the chondrogenic culture system we use currently has a strong effect on promoting ECM production, it reduces chondrocyte survival, which is obviously unfavorable for the stability and maintenance of regenerated cartilage in the long term. Although JNK inhibitors were shown to reduce chondrocyte apoptosis in a model of OA (Lu et al., 2014), whether JNK inhibitors can promote chondrocyte proliferation and reverse the negative effect of current chondrogenic system on cell survival in 3D cartilage regeneration models is still unclear. Here, we found that SP600125 promoted the proliferation of chondrocyte at both cellular and tissue levels. More importantly, adding SP600125 reversed the negative effect of CM on cell survival in our 3D culture system, improved the survival of chondrocytes, and thus provided a good cell basis for subsequent cartilage regeneration.

In addition to the active chondrocytes, cartilaginous ECM metabolism is a key factor in determining the quality of regenerated cartilage. A balance between anabolism and catabolism of the ECM is vital for the long-term survival and stability of cartilage (Karsdal et al., 2016; Huang et al., 2018; Iturriaga et al., 2018). When dominated by anabolism, such as the *in vitro* cartilage regeneration models, cartilage tends to grow; when dominated by catabolism, such as in models of OA, cartilage tends to degrade. JNK inhibitors could significantly reduce the cartilaginous degradation associated with OA by inhibiting the activity of MMP13 (Ge et al., 2017). However, the effects of JNK inhibitors on cartilage ECM metabolism in 3D cartilage regeneration models are still unclear. As expected, our results demonstrate that JNK inhibitors had a markedly inhibitory effect on ECM degradation, evidenced by significant downregulation of ECM catabolism-related genes such as those encoding *TNF-*α, *IL-1*β, and *MMP13* at both cellular and tissue levels. According to previous reports, it was demonstrated that OA chondrocytes produced matrix-degrading enzymes primarily MMP13 in response to pro-inflammatory cytokines such as TNF-α and IL-1, which promoted the breakdown of articular cartilage (Kimura et al., 2009; Akutsu et al., 2013; Zhang et al., 2016). Therefore, we speculate that the inhibitory effect on the expression of *MMP13* was a response to the reducing expression of pro-inflammatory cytokines such as *TNF-*α and *IL-1*β and thus lead to the inhibition of ECM degradation. Given that reducing ECM degradation is a vital factor in cartilage survival and maintenance, SP600125 had a positive effect on ECM deposition during *in vitro* cartilage regeneration.

The effect of JNK inhibitors on ECM anabolism is another important issue. Unexpectedly, ECM synthesis was also greatly inhibited at both the cellular level and our 3D cartilage regeneration model, as indicated by SEM, histology, quantitative evaluations of ECM, and the expression levels of cartilage-related genes. According to our results, the inhibitory effect of SP600125 on ECM synthesis might result from reduced levels of *TGF-*β*1* and *IGF-1*. This would greatly reduce the self-secretion of these anabolic factors and thus inhibit ECM synthesis (Sandell and Aigner, 2001). Because ECM anabolism plays a dominant role during cartilage regeneration *in vitro*, SP600125 alone was obviously unfavorable for cartilage regeneration in terms of inhibiting ECM synthesis.

Fortunately, this could be canceled and even reversed by combining supplementation with a chondrogenic culture system. According to our results, the combination of SP600125 and CM achieved better ECM synthesis and more mature cartilage regeneration compared with standard medium, as indicated by histology, quantitative evaluations of ECM, and the expression levels of cartilage-related genes. The inhibitory effect of SP600125 on ECM synthesis could be reversed, and this mainly contributed to the high contents of exogenous TGF-β1 and IGF-1 in the chondrogenic culture system. Obviously, the addition of exogenous TGF-β1 and IGF-1 reversed the downregulation of these factors caused by SP600125 and thus kept high contents of anabolic factors in the culture system, which is the key factor for satisfactory cartilage regeneration. More importantly, the addition of SP600125 significantly enhanced the numbers of active cells and greatly inhibited ECM degradation, which effectively made up for the deficiencies of our current chondrogenic culture system in regulating cell survival and ECM catabolism. Therefore, the combination of SP600125 and CM not only had no negative effect on ECM deposition but also helped to achieve better *in vitro* cartilage regeneration in terms of enhancing cell survival and inhibiting ECM catabolism.

In summary, using an *in vitro* 3D cartilage regeneration model, here, we demonstrated that the combination of a JNK inhibitor and CM could efficiently improve the quality of *in vitro* cartilage regeneration by promoting cell proliferation, reducing ECM degradation, and enhancing ECM synthesis. Although some issues such as dosage and the duration of SP600125 supplementation need to be further investigated for future clinical applications, this study suggests a feasible strategy for optimizing chondrogenic culture systems and achieving satisfactory *in vitro* cartilage regeneration.

## Data Availability Statement

The datasets presented in this study can be found in online repositories. The names of the repository/repositories and accession number(s) can be found in the article/Supplementary Material.

## Ethics Statement

The animal study was reviewed and approved by Animal Care and Experiment Committee of Shanghai Jiao Tong University School of Medicine.

## Author Contributions

PZ: conception and design, collection and assembly of data, data analysis and interpretation, and manuscript writing. YaL: provision of study material and collection and assembly of data, and data analysis and interpretation. LJ and ZC: provision of study material, collection and assembly of data. WZ and YuL: conception and design and data analysis and interpretation. JC: conception and design, administrative support, financial support, manuscript writing, and final approval of manuscript. YC: conception and design, administrative support, and final approval of manuscript. GZ: conception and design, administrative support, financial support, manuscript writing, and final approval of manuscript. All authors contributed to the article and approved the submitted version.

## Conflict of Interest

GZ, YC, and YuL were employed part-time by the company National Tissue Engineering Center of China. The remaining authors declare that the research was conducted in the absence of any commercial or financial relationships that could be construed as a potential conflict of interest.
