# Supplementary material for: SP600125, a JNK-Specific Inhibitor, Regulates in vitro Auricular Cartilage Regeneration by Promoting Cell Proliferation and Inhibiting Extracellular Matrix Metabolism
Source: Front Cell Dev Biol. 2021 Mar 16;9:630678. doi: 10.3389/fcell.2021.630678 (PMC8010669; doi:10.3389/fcell.2021.630678)
Supplement: Supplementary file 1 [file Table_1.DOCX]

Supplementary Material

# Supplementary Figures and Tables

## Supplementary Figure 1


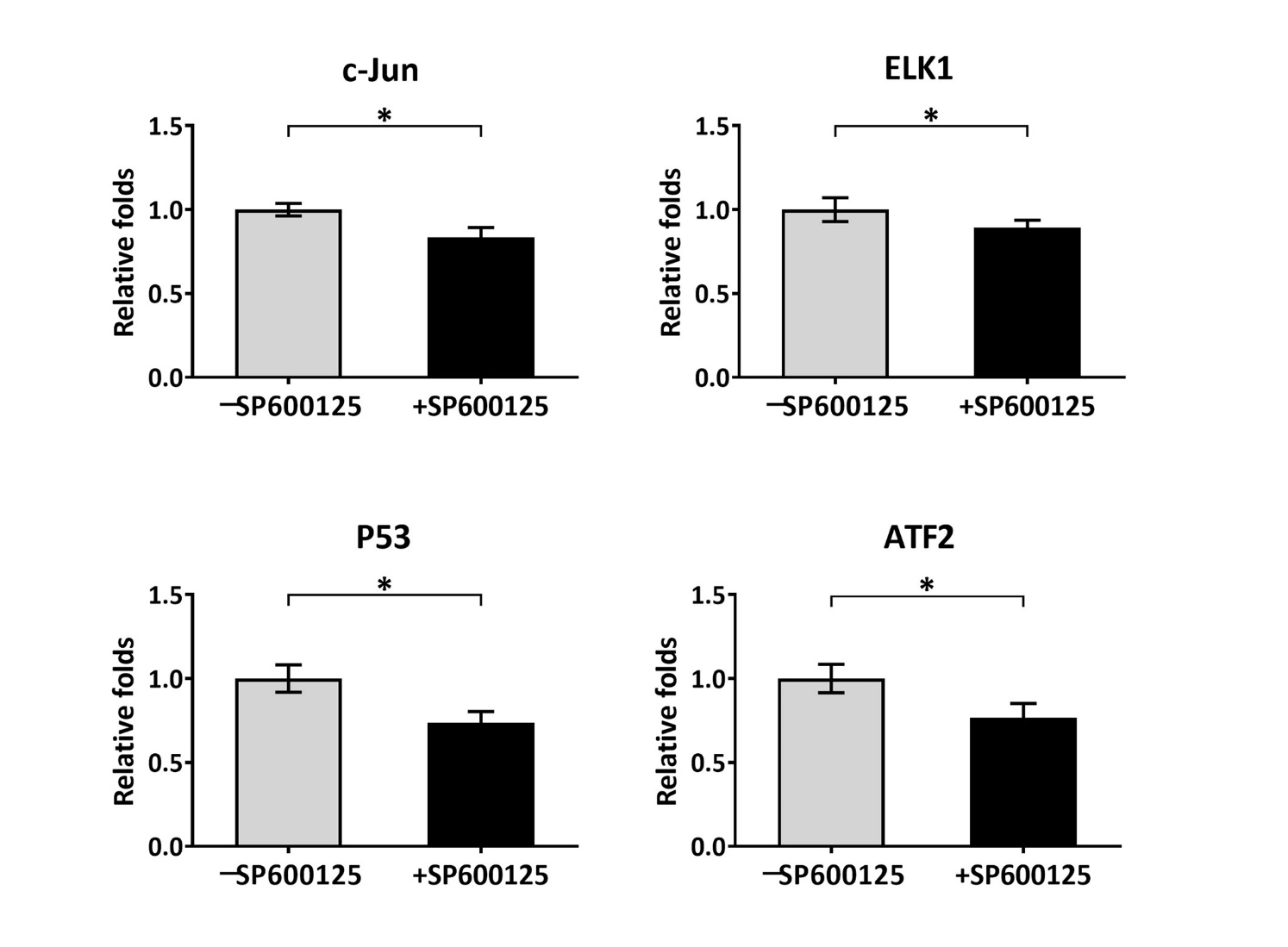


**Supplementary Figure 1.** JNK signaling pathway related genes analysis of chondrocytes. After treating with SP600125 for 24h, expression of JNK related genes (c-Jun, ELK1, ATF2 and P53) were decreased, confirming that SP600125 did had an inhibitory effect on JNK signaling pathway.

## Supplementary Figure 2


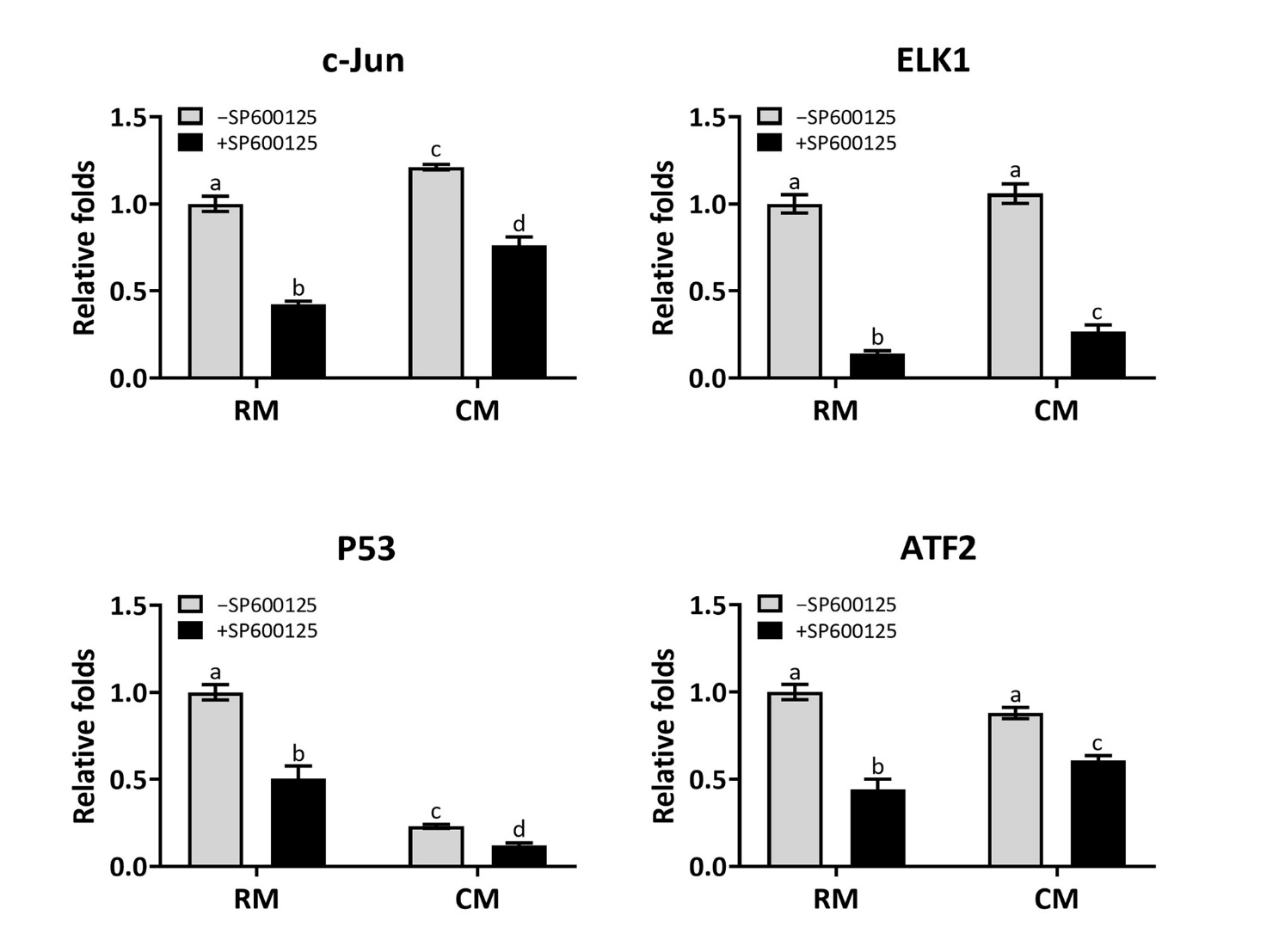


**Supplementary Figure 2.** JNK signaling pathway related genes analysis of in-vitro cultured cartilages. After the samples were cultured in vitro for 8 weeks, the expression levels of JNK-related genes (c-Jun, ELK1, ATF2 and P53) in +SP600125 groups were significantly decreased, confirming that SP600125 did had an inhibitory effect on JNK signaling pathway.

## Supplementary Table 1

**Supplementary Table 1.** Forward and reverse primer sequences of genes that were analyzed.
